# An Easy and Reliable Strategy for Making Type I Interferon Signature Analysis Comparable among Research Centers

**DOI:** 10.3390/diagnostics9030113

**Published:** 2019-09-04

**Authors:** Alessia Pin, Lorenzo Monasta, Andrea Taddio, Elisa Piscianz, Alberto Tommasini, Alessandra Tesser

**Affiliations:** 1 Department of Medicine, Surgery and Health Sciences, University of Trieste, 34127 Trieste, Italy (A.P.) (A.T.); 2 Clinical Epidemiology and Public Health Research Unit, Institute for Maternal and Child Health—IRCCS “Burlo Garofolo”, 34137 Trieste, Italy; 3 Department of Paediatrics, Institute for Maternal and Child Health—IRCCS “Burlo Garofolo”, 34137 Trieste, Italy; 4 Department of Advanced Diagnostic and Clinical Trials, Institute for Maternal and Child Health—IRCCS “Burlo Garofolo”, 34137 Trieste, Italy (E.P.) (A.T.)

**Keywords:** interferon signature score, inter-laboratory variability, data sharing, systemic lupus erythematosus, interferonopathies, biostatistics

## Abstract

Interferon-stimulated genes (ISGs) are a set of genes whose transcription is induced by interferon (IFN). The measure of the expression of ISGs enables calculating an IFN score, which gives an indirect estimate of the exposition of cells to IFN-mediated inflammation. The measure of the IFN score is proposed for the screening of monogenic interferonopathies, like the Aicardi-Goutières syndrome, or to stratify subjects with systemic lupus erythematosus to receive IFN-targeted treatments. Apart from these scenarios, there is no agreement on the diagnostic value of the score in distinguishing IFN-related disorders from diseases dominated by other types of cytokines. Since the IFN score is currently measured in several research hospitals, merging experiences could help define the potential of scoring IFN inflammation in clinical practice. However, the IFN score calculated at different laboratories may be hardly comparable due to the distinct sets of IFN-stimulated genes assessed and to different controls used for data normalization. We developed a reliable approach to minimize the inter-laboratory variability, thereby providing shared strategies for the IFN signature analysis and allowing different centers to compare data and merge their experiences.

## 1. Introduction

Type I interferon (IFN) production is part of the innate immune response to viruses or intracellular bacteria, which is triggered by the sensing of pathogen-associated nucleic acids [1]. Even though the identification of IFNs dates back to the 50s–60s [2], the description of a group of mendelian disorders with dysregulated IFN-mediated inflammation has only recently shed light on the fine regulation of the production and action of these cytokines [3]. Of note, this new group of disorders, known as type I interferonopathies, displays significant phenotypic overlaps with both systemic lupus erythematosus (SLE) and congenital viral infections of the TORCH (Toxoplasmosis, Rubella, Cytomegalovirus, Herpes simplex) and HIV (human immunodeficiency virus) groups [3,4]. 

Type I interferonopathies are marked by the hyper-expression of a set of genes (IFN-stimulated genes, ISGs) in inflamed tissue and often in peripheral blood, leading to the definition of the so-called “IFN signature” [5,6]. The IFN signature was firstly defined in subjects with SLE to assess the level of IFN related inflammation and to help stratify patients to receive IFN targeted treatments [7,8,9].

Since then, the measure of expression of the ISGs (IFN signature analysis) is increasingly used in biomedical research centers, as well as for the functional classification of other conditions characterized by a type I IFN dysregulation [10], to distinguish such conditions from classical inflammatory disorders predominantly mediated by other cytokines, like Tumor Necrosis Factor α and Interleukin 1 (i.e., inflammatory bowel diseases, rheumatoid arthritis, and periodic fevers) [11].

Different ISG sets were identified to evaluate interferon-mediated autoinflammation and are frequently restricted to 5-6 targeted genes [6,10,12,13], suitable, for example, for the discrimination of Aicardi-Goutières syndrome (AGS) [6,10]. However, IFN signature analysis could be extended to larger gene lists [7] or even restricted to just one single gene, particularly when directly assessed in affected tissues, as in the case of dermatomyiositis [14,15,16]. 

Even though the IFN signature measure has become widely available at research hospitals, there is no consensus for the selection of calibration controls. Thus, it is hard to compare data among distinct centers and to estimate the potential of IFN signature testing for discriminating among inflammatory conditions in the clinical practice. For example, thousands of subjects with antiphospholipid syndrome have been described in multicenter studies [17], while the interferon score has been separately studied in several small series without allowing the merging of results [18,19,20,21,22].

The main problem hindering the use of IFN signature analysis for in vitro diagnostics is the expression of data relativized to independent healthy control(s) in each laboratory, leading to unpredictable inter-laboratory variability. It is logical to assume that the use of pooled cDNA from healthy donors can represent a convenient strategy for calibrating Real Time quantitative PCR (qPCR) for the assessment of the IFN score. However, it can be difficult to predict the optimal number of samples to be pooled, which requires minimizing variability between one pool and another prepared at distinct laboratories, as already pointed out by others [23,24].

This study aims to investigate, through laboratory, bioinformatic, and statistical analyses, a reliable approach to minimize the variability that can be observed in inter-laboratory assays. 

The final goal is providing shared recommendations for IFN signature analysis and interpretation of data in the clinical practice, thereby allowing data sharing among reference centers and improving knowledge on IFN-related disorders.

## 2. Materials and Methods 

The study is part of the IRCCS Burlo Garofolo project RC #24/2017, approved by the Institutional Review Board and by the Friuli Venezia Giulia Independent Ethical Committee (2018-SPER-079-BURLO, N. 0039851, approved on 12 December 2018). All investigations were performed after obtaining written informed consent from volunteers and patients or their parents/guardians. 

### 2.1. Subjects

On wet IFN signature analysis was assessed by quantitative PCR (qPCR) in ten young-aged healthy subjects (Dataset A, ten out of eleven individuals, five males and five females).

To establish whether data from qPCR and RNAseq analysis were comparable, IFN signature on wet (by qPCR) and in silico (by RNAseq) analysis was performed in twenty subjects with inflammatory diseases, such as systemic lupus erythematosus (SLE), interferonopathies or inflammatory bowel diseases (IBD), and patients’ relatives, recruited at our center (Dataset E). A brief description of patients’ clinical diagnosis is displayed in Table S1.

To increase healthy donors numerosity, in silico IFN signature investigation (by RNAseq) has been performed in twenty healthy individuals, collected at our center Dataset A (four out of eleven individuals), and selected from different whole blood RNA-sequencing (RNAseq) open-access web-based datasets: we sorted another three datasets, while considering exclusively healthy control samples, accessible at ArrayExpress (accession number E-MTAB-5735, Dataset B, five individuals) and at the Gene Expression Omnibus (GEO) (accession number GSE112057, Dataset C, nine individuals; GSE90081, Dataset D, two individuals). Specification about gender was not available for all the samples. However, sex has been easily inferred by expression analysis of the sex-specific genes *RPS4Y1* and *USP9Y*. 

The dataset composition is shown in Table 1.

### 2.2. Sample Collection, RNA Isolation and cDNA Preparation

Peripheral blood was collected in PAXgene Blood RNA Tubes (PreAnalytiX, Hombrechtikon, Switzerland) and, after two-hours incubation at room temperature, tubes were frozen at −20 °C until processing. Total RNA was extracted with PAXgene Blood RNA Kit (PreAnalytiX, Switzerland), following the manufacturer’s instructions, and quantified with NanoDrop Spectrophotometer (Thermo Fisher, Waltham, MA, USA). RNA integrity was checked using an Agilent Technologies 2100 Bioanalyzer. 

Up to 1 μg of total RNA was retro-transcribed using SensiFAST cDNA Synthesis Kit (Bioline, London, UK). 

### 2.3. IFN Signature Analysis

The expression of six IFN-stimulated genes was assessed by qPCR using AB 7500 Real Time PCR System (Applied Biosystems, Waltham, MA, USA), TaqMan Gene Expression Master Mix (Applied Biosystems, USA) and UPL Probes (Roche, Basel, Switzerland) for *IFI27*, *IFI44L*, *IFIT1*, *ISG15*, *RSAD2*, and *SIGLEC1*. Using AB 7500 Real Time PCR software, each target quantity was normalized with the expression level of *HPRT1* and *G6PD*, and the relative quantification (RQ) was conducted relating to a “calibrator” sample (mix of ten healthy controls, Dataset A) using the 2^−ΔΔCt^ method [25]. The median fold change of the six genes was used to calculate the IFN score for each patient.

### 2.4. RNAseq Analysis

Transcriptome sequencing was performed using the TruSeq Stranded mRNA Sample Preparation kit (Illumina, San Diego, CA, USA) and sequenced on a NovaSeq 6000 platform (Illumina, San Diego, CA, USA), generating 2X100 bp paired-end reads (30 million reads per sample) in twenty subjects from Dataset E (patients and patients’ relatives) and four out of eleven controls from Dataset A.

RNAseq raw data (either our data and open-access web-based data) workflow was conducted as follows: quality control by FastQC (https://www.bioinformatics.babraham.ac.uk/projects/fastqc/), quality filtering by Trim Galore (https://www.bioinformatics.babraham.ac.uk/projects/trim_galore/), read alignment to hg38 using annotation from GENECODE v.31 (https://www.gencodegenes.org/) with STAR [26], reads counting into genes by featureCounts [27].

Data of patients with autoinflammatory diseases and three healthy individuals of our dataset were normalized and analyzed for differentially expressed genes by DESeq2 [28]. From the result table, we only considered the ISGs and evaluated their relative fold changes on each patient compared to the set of controls.

To assess the ISGs expression variability within the group of twenty healthy subjects, shortlisted from the datasets described above, we determined the expression values for each gene, normalized by Fragments Per Kilobase per Million mapped reads (FPKM) method with edgeR (*rpkm* function) [29,30], using the values from the “Length” column, in the featureCounts’ output, for the calculation.

Principal component analysis (PCA), useful for data visualization, was conducted with DESeq2, to define the overall variability between samples.

### 2.5. Statistical Analyses

Considering that each of the six genes measured was expressed on a different scale, we decided to calculate the sample size based on the coefficient of variation, instead than the mean and the standard deviation. We further hypothesized that different runs did not increase the variation in comparing the samples, assuming the only origin of variability to be represented by the subjects’ heterogeneity. 

To determine the statistical power for data obtained by qPCR and RNAseq, we computed the noncentrality parameter (λ) using GPower 3.1.9.2. software [31,32], with a generic two-tailed *t*-test, given α = 0.05, β = 0.2, and degrees of freedom equal N-1. If the noncentrality parameter under these conditions (reference value, “λref”) resulted in being lower than the one calculated on our samples, we considered the sample size as appropriate. 

GraphPad Prism 6 software was employed for χ^2^ contingency analysis; *p*-values <0.05 were considered significant. 

To identify the appropriate sample size for variability assessment, we computed λ for increasing numerosity (up to forty) using GPower 3.1.9.2. (Heinrich-Heine University Düsseldorf, Germany), and determined a “plateau value” by an exponential decay function (GraphPad Prism 6 software, La Jolla California USA). 

## 3. Results

### 3.1. Variability Assessment in IFN-Stimulated Genes Expression in Healthy Controls (Dataset A) Analyzed by qPCR 

**Figure 1:**
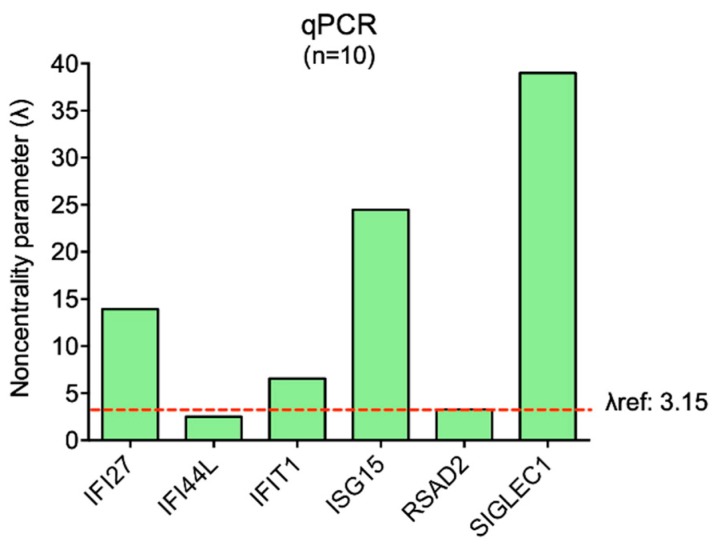
Graphical representation of the noncentrality parameter (λ) calculated for each ISG by on wet qPCR analysis. The λref for ten subjects is displayed by the dashed line and reported in the figure. Sample size is considered as appropriate when λ computed on each gene is higher than λref.

The variability of expression of the six ISGs (*IFI27*, *IFI44L*, *IFIT1*, *ISG15*, *RSAD2*, *SIGLEC1*) was assessed in ten healthy controls processed by on wet qPCR analysis. Five out of six genes showed low variability coefficients and noncentrality parameters (λ) that fulfilled the analysis criteria (as described in Materials and Methods, Section 2.5) (Table 2). Only *IFI44L* did not comply with the analysis parameters, presenting higher variability and a lower λ than the reference value (λref) (Figure 1)

Thus, ten healthy controls could not be considered an appropriate sample size to represent an ideal healthy population, in which the physiologically floating expression values of the ISGs present acceptable variability. For this reason, we should increase the numerosity of healthy controls to obtain a suitable pool in which the gene expression variability is minimized. 

### 3.2. IFN-Stimulated Genes Expression Evaluated by qPCR or RNAseq Analysis Are Comparable

To improve the power of the variability measurement, we decided to take advantage of RNAseq open-access web-based data, as an easy source to increase the number of healthy subjects to calculate ISGs interindividual differences. This choice came from comparisons between the relative ISGs fold change assessed in the same twenty subjects (Dataset E) by both qPCR and RNAseq analysis, selecting the same set of three out of eleven healthy controls from Dataset A, to normalize data for both techniques. 

**Figure 2:**
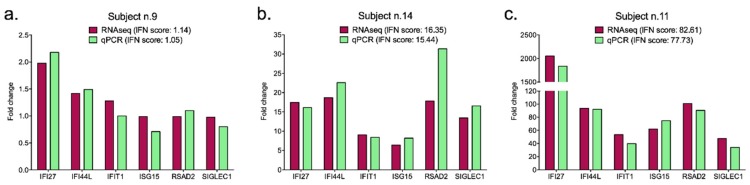
Representative display of low (**a**), intermediate (**b**) and high (**c**) IFN signatures analyzed by both qPCR and RNAseq for each subject. For the optimal graphical representation of all histograms, the scales of values are set different. The IFN scores computed for each subject are reported in the legend of the figures.

Some subjects showed different relative expression values for the same gene calculated by on wet qPCR and in silico RNAseq, but the overall results of the IFN signatures (IFN scores) were extremely consistent between the two techniques for each individual (χ^2^ contingency analysis *p*-value = 0.405, not significant). The comparability of IFN scores is easily explained, considering that these values represent the median of the six relative ISGs quantifications, and they broadly reflect the overexpression status in the analyzed sample (Table 3). Thus, the two methods provided the same trend in gene expression in subjects presenting low, intermediate, and high IFN signatures, as indicated by the three representative graphs in Figure 2 (Subject n.9: low IFN signature; Subject n.14: intermediate IFN signature; Subject n.11: high IFN signature).

### 3.3. Preliminary Analysis for Sample Selection and Variability Assessment in IFN-Stimulated Genes Expression Analyzed by RNAseq 

Given the comparability of data between qPCR and RNAseq, we exploited the availability of large open-access web repositories containing RNAseq data to calculate the proper sample size for the assessment of the IFN score with an acceptable inter-laboratory variability. 

**Figure 3:**
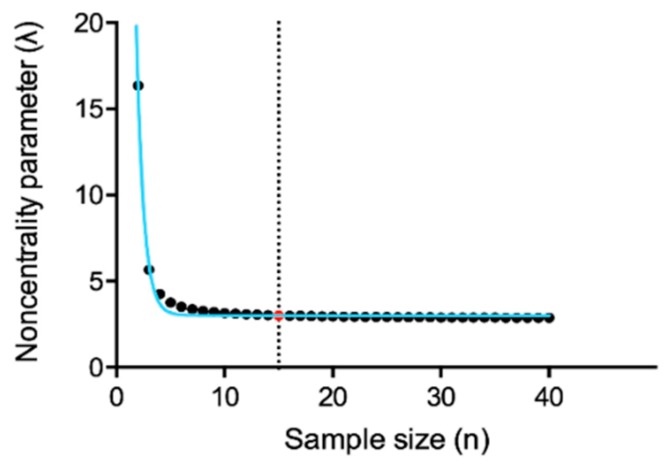
Plateau value (reported as a red dot) threshold (reported as a dotted vertical line) of λ computed for sample sizes up to forty subjects. Each sample size is represented by a black dot. In blue the exponential decay function curve of the previously computed λ.

To select the appropriate sample size, we firstly calculated the noncentrality parameter (λ) on a numerosity up to forty individuals using GPower software. Then, we determine the plateau value of the exponential decay function of the previously computed λ values. The provided plateau (3.01) corresponds to λ for sample size *n* = 15 (Figure 3), leading us to consider fifteen subjects as an appropriate sample size. For more experimental strength, we considered both *n* = 15 and *n* = 20 in the following analyses. We did not further increase the sample size over twenty subjects, whereas exceeding this number might bring difficulties in term of donors’ collection. 

We performed a principal component analysis (PCA), a data visualization analysis, to evaluate the total expression variation of the six ISGs and to define the most homogeneous set of twenty healthy individuals. This investigation allows the detection of possible rare outliers with the highest variance that might not be considered as suitable controls to study IFN signature.

RNAseq records have been chosen considering the presence of similar features such as blood collection type, RNA extraction protocol and library selection, to reduce as much as possible the technical procedure variability (Table 3). 

**Figure 4:**
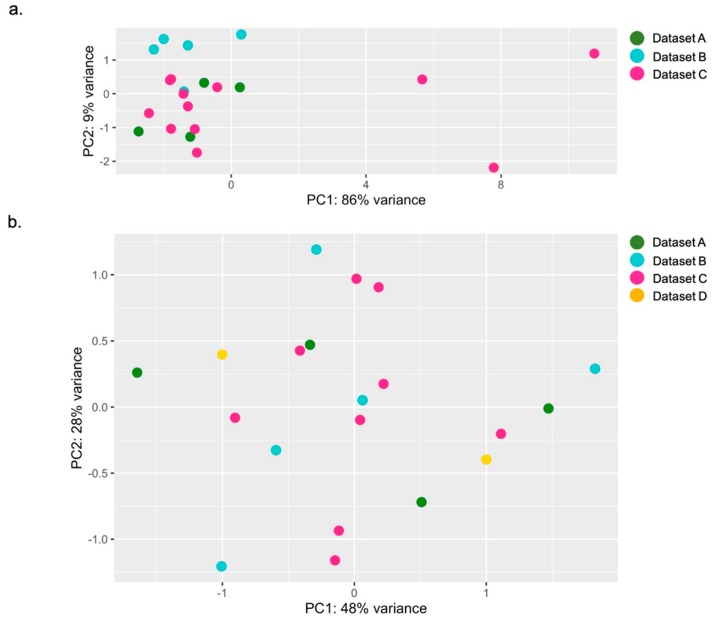
Overall ISGs expression variance between individuals. PCA of the first selection (**a**), with evidence of the three outliers from Dataset C, and of the final twenty-controls-sized group (**b**), showing twenty samples uniformly distributed (4 from Dataset A, 5 from Dataset B, 9 from Dataset C and 2 from Dataset D).

As a first attempt, we investigated all the RNAseq samples of healthy donors from Dataset A (data from our center, *n* = 4/11), Dataset B (E-MTAB-5735, *n* = 5) and Dataset C (GSE112057, *n* = 12), twenty-one specimens in total. Figure 4a displays the PCA results showing the overall ISGs expression variability between individuals. The analysis exhibited a higher variance in three out of twenty-one subjects: we thus decided not to include these three samples in further studies, collecting eighteen samples that were suitable for our purpose. To get the proper numerosity (*n* = 20), we examine Dataset D (GSE90081, *n* = 12) and we ran the same analysis again, obtaining a satisfactory level of variation, without outliers, between datasets and among individuals. From these preliminary observations, we randomly chose two out of twelve samples from Dataset D, combining them with data previously selected. Again, we observed an acceptable gene expression variability among our final twenty-controls-sized group (Table 4), as shown in Figure 4b. 

**Figure 5:**
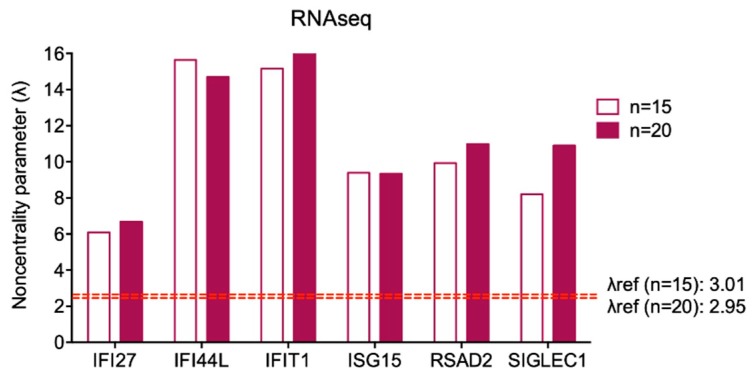
Graphical representation of λ calculated for each ISG by in silico RNAseq analysis. The λrefs for fifteen and twenty subjects are displayed by the red dashed line and reported in the figure. Sample size is considered appropriate when λ computed on each gene is higher than λref.

We calculated the λref for larger samples of healthy controls (fifteen and twenty subjects) using GPower software, and the variability of the ISGs expression in fifteen and twenty healthy controls processed by in silico RNAseq analysis. Table 5 shows that all the variability coefficients are considerably low and all λ calculated fulfilled the analysis parameters (Figure 5) (expression values of single gene for each control are displayed in Table S2. Thus, we can hypothesize that pooling together samples from fifteen or twenty healthy subjects could also be considered a proper sample size in qPCR analyses. 

### 3.4. Pooling Twenty Subjects Could Be Considered an Optimal Strategy to Minimize Gene Expression Variability among Healthy Controls for on Wet IFN Signature Analysis 

We checked whether the values of mean, SD and variability coefficient obtained by on wet qPCR on ten controls met the λ criteria considering *n* = 15 and *n* = 20 subjects as already calculated, assuming that the variability coefficient does not change as the sample size increases (Table 6).

**Figure 6:**
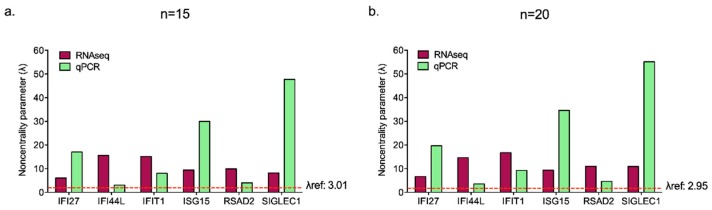
Graphical representation of λ calculated for each ISG by in silico RNAseq analysis and hypothesized qPCR assessment considering fifteen (**a**) and twenty (**b**) subjects. The λrefs values are displayed by the red dashed line and reported in the figure. Sample size is considered appropriate when λ computed on each gene is higher than λref.

The analysis provides quite good results for both sample sizes tested, even if *IFI44L* showed a λ (3.08) very close to λref (3.01). For this reason, we can consider more adequate to increase the numerosity to twenty subjects, a still easy-to-gather number of healthy donors. Thus, we could assert that twenty healthy controls could be considered a suitable sample size for IFN signature analysis performed by qPCR, as predicted by in silico RNAseq (Figure 6).

## 4. Discussion

The clinical employment of the IFN signature is strictly related to the screening of pathological conditions characterized by type I IFN dysregulation [24]. However, several studies have been carried out to associate the IFN-related inflammation with specific clinical or laboratory features of rheumatologic conditions, like systemic lupus erythematosus, primary antiphospholipid syndrome, Sjögren syndrome, rheumatoid arthritis, autoimmune myositis and systemic sclerosis [18,20,33,34,35,36,37,38]. Some Authors proposed that the assessment of IFN inflammation may help identify subgroups of patients with a better response to specific treatments, as B cell targeted therapies [39,40,41,42]. Moreover, since most anti-inflammatory or immunomodulatory agents have only a weak effect on IFN inflammation, the calculation of the IFN score may also serve to guide targeted therapy approaches with novel drugs like Janus Kinase inhibitors [10]. 

However, there is no consensus on a shared and validated method to classify different inflammatory conditions by transcriptome analysis. Crow and collaborators used pooled cDNA from healthy donors as calibrator for qPCR, and after assessing a large number of healthy controls and patients with Aicardi-Goutières Syndrome calculated a fixed cut-off value of normality suitable for the screening of interferonopathies [6]. However, this cut-off has been validated to facilitate the detection of monogenic interferonopathies and not to assess IFN inflammation in other conditions. Moreover, the reference to locally pooled control cDNA may make it difficult to compare results obtained in distinct centers. 

Even though multiple ISG panels have been described either in peripheral blood cells or affected tissues, many laboratories set their assays by analyzing a minimal set of 5-6 targeted-genes, usually including the set proposed by Crow et al. This set has been applied to thousands of analyses and its potential for screening of monogenic interferonopathies is established. After the normalization of results on at least two housekeeping genes, the main source of variability limiting interlaboratory comparison of data consists in the use of different controls for data normalization. Indeed, this is a remarkable problem, considering that the potential role of IFN signature analysis in clinical practice can be defined only by sharing data among research centers, comparing or merging case series. Of course, the best option for the future should rely on the development of industrially manufactured kits validated for In Vitro Diagnostics (IVD) and usable worldwide with the same reference values, not only in the most advanced research areas. Conversely, only the analysis and comparison of data available at various centers can tell industries whether the development of such diagnostic kits is worthwhile or not. Thus, we focused on a strategy that could be immediately applied in biomedical laboratories to facilitate the sharing of experience and minimize the inter-laboratory variability. 

We investigated if using a pool of biological samples from healthy controls could solve the data normalization issue, which is the main source of inter-laboratory variability. We proposed that this strategy could “equalize” the differences in gene expression that physiologically occur among individuals.

For this purpose, we evaluated if any pool of healthy controls more than a given number *n* of subjects could be suitable to level differences, through an approach based on laboratory (qPCR), bioinformatic (RNAseq), and statistical data integration analyses. 

Starting from the ISGs expression assessment in peripheral blood from ten healthy subjects by on wet qPCR analysis, we found that this sample size is not suitable for equalizing the variable expression levels of all the six ISGs in healthy volunteers. To find the appropriate number of samples to be pooled together with low enough variability, we further investigated if public data from RNAseq can be exploited to expand our analysis. We thus compared relative ISGs fold change values calculated by qPCR and RNAseq analysis for the same subjects with the same calibrator, showing that the two methods yield comparable results with very low variability between each other. Previous studies compared gene expression measurements generated by in silico RNAseq and on wet qPCR assays, showing consistent results between the two methods for most genes, with the only exception of some genes generally characterized by small size and low expression levels. None of the ISGs is included in the list of genes with inconsistent estimation of expression according to the Authors [43]. Our results confirmed that RNAseq and qPCR generate consistent results in the assessment of ISGs expression levels. Thus, based on the literature and on our preliminary results, we considered RNAseq as a good asset to increase the number of healthy subjects on which to calculate interindividual differences in ISGs expression, exploiting both RNAseq samples available at our center and open-access web-based data.

The results of our study support the choice of pooling twenty healthy controls for the normalization of the assay, allowing to express results as relative to a “standard set of controls”. Of note, we validated this sample size only for the selected set of six ISGs in peripheral blood cells. The expression of other genes included in larger panels, may present higher variability among donors and between different affected tissues. However, the same procedure that we have described can also be used to define the optimal sample size for other transcription profiles, for interferonopathies or for other rheumatologic disorders. The performance of the proposed twenty-controls-sized standard could be improved by analyzing all the donors separately before pooling, and by performing cluster variance analysis, which can enable excluding rare donors with outlier variance.

## Figures and Tables

**Table 1:** Dataset composition: subjects, size, methods and purposes.

Datasets	Subjects	*n* Total (F/M)	Method (*n*)	Purpose
A—Data of from our center; Accession: #	Healthy donors	11 (5/6)	qPCR (10) RNAseq (3)	To test the variability of expression of the six ISGs in a healthy donor small group available at out center
			RNAseq (1)	To increase the healthy donor group size, to improve the power of the variability measurement
B—Accession: E-MTAB-5735	Healthy donors	5 (2/3)	RNAseq (5)	To increase the healthy donor group size, to improve the power of the variability measurement
C—Accession: GSE112057	Healthy donors	12 (6/6)	RNAseq (9)	To increase the healthy donor group size, to improve the power of the variability measurement
D—Accession: GSE90081	Healthy donors	12 (12/0)	RNAseq (2)	To increase the healthy donor group size, to improve the power of the variability measurement
E—Patients and patient’s relatives recruited at our center	Patients	20 (9/11)	qPCR (20) RNAseq (20)	To compare IFN signature results between qPCR and RNAseq analyses

# data not present in open-access web-based datasets.

**Table 2:** Variability assessment for interferon-stimulated genes (ISGs) expression values quantified in ten out of eleven healthy subjects from Dataset A by qPCR.

	IFI27	IFI44L	IFIT1	ISG15	RSAD2	SIGLEC1
Mean	4.57	0.72	2.18	4.61	1.24	5.14
SD	1.04	0.91	1.05	0.60	1.20	0.42
Variability coefficient	0.23	1.26	0.48	0.13	0.96	0.08
λ (*n* = 10)	13.92	**2.52**	6.56	24.47	3.28	38.99
λref: 3.15						

SD: standard deviation; λref: noncentrality parameter (λ) calculated based on α = 0.05, β = 0.2, and degrees of freedom equal N-1. Sample size is considered as appropriate when λ computed on each gene is higher than λref. The value below λref is highlighted in bold.

**Table 3:** Comparison of interferon (IFN) scores determined in twenty subjects by both qPCR and RNAseq by χ^2^ contingency analysis (*p*-value = 0.405, not significant).

Subject *n*.	IFN Score	
	In Silico RNAseq	On Wet qPCR
1	3.26	5.01
2	6.79	10.05
3	7.12	9.85
4	8.58	10.74
5	1.55	1.37
6	1.11	0.97
7	0.55	0.67
8	0.22	0.19
9	1.14	1.05
10	3.68	2.60
11	77.73	82.61
12	44.03	94.25
13	17.44	16.48
14	15.44	16.35
15	37.43	49.98
16	37.44	84.99
17	1.07	1.18
18	2.39	4.70
19	3.01	1.71
20	1.82	1.82
Mean	19.83	13.59
SD	31.19	20.33
Variability coefficient	1.57	1.50

SD: standard deviation.

**Table 4:** Detailed report of final twenty-controls-sized healthy subject groups.

Datasets	Subjects		RNAseq Details		
Authors & Accession	Female (*n* = 9)	Male (*n* = 11)	Whole Blood collection/RNA extraction	RNAseq library preparation/platform	Read Length
A—Data from our center; Accession:#	2	2	PAXgene blood RNA tube/PAXgene Blood RNA Kit	Illumina TruSeq stranded mRNA library protocol/Novaseq	Paired-end 100 bp reads
B—Rodero MP, et al., 2017; Accession: E-MTAB-5735	2	3	PAXgene blood RNA tube/PAXgene Blood RNA Kit	Illumina TruSeq stranded mRNA library protocol/Illumina HiSeq 2000	Paired-end 75 bp reads
C—Mo A., et al., 2018; Accession: GSE112057	3	6	Tempus Tube/Tempus Spin isolation RNA kit	Illumina TruSeq stranded mRNA library protocol/Illumina HiSeq Rapid Run	Paired-end 100 bp reads
D—Shchetynsky K., et al., 2017; Accession: GSE90081	2	-	PAXgene blood RNA tube/PAXgene Blood miRNA kit	Standard illumina TruSeq RNA protocol, following PolyA enrichment/Illumina HiSeq 2000	Paired-end 100 bp reads

# data not present in open-access web-based datasets.

**Table 5:** Variability assessment for IFN-stimulated genes expression values evaluated in fifteen and twenty healthy subjects by in silico RNAseq analysis.

		IFI27	IFI44L	IFIT1	ISG15	RSAD2	SIGLEC1
*n* = 15	Mean	0.35	1.14	5.06	30.17	2.21	1.17
	SD	0.23	0.28	1.29	12.44	0.86	0.55
	Variability coefficient	0.64	0.25	0.26	0.41	0.39	0.47
	λ	6.09	15.64	15.16	9.39	9.93	8.21
	Λref: 3.01						
*n* = 20	Mean	0.33	1.26	5.17	30.09	2.47	1.22
	SD	0.22	0.38	1.38	14.41	1.00	0.50
	Variability coefficient	0.67	0.30	0.27	0.48	0.41	0.41
	λ	6.68	14.70	16.77	9.34	10.98	10.90
	λref: 2.95						

SD: standard deviation; λref: noncentrality parameter calculated based on α = 0.05, β = 0.2, and degrees of freedom equal N-1. Sample size is considered as appropriate when λ computed on each gene is higher than λref.

**Table 6:** Estimated variability assessment for ISGs expression values in fifteen and twenty healthy subjects for qPCR analysis assuming the same variability coefficient for increasing sample size.

	IFI27	IFI44L	IFIT1	ISG15	RSAD2	SIGLEC1
Mean	4.57	0.72	2.18	4.61	1.24	5.14
SD	1.04	0.91	1.05	0.60	1.20	0.42
Variability coefficient	0.23	1.26	0.48	0.13	0.96	0.08
λ (*n* = 15)	17.05	3.08	8.04	29.97	4.02	47.75
λref: 3.01						
λ (*n* = 20)	19.69	3.56	9.28	34.61	4.64	55.14
λref: 2.95						

Mean, standard deviation (SD) and variability coefficient values previously determined on ten subjects are reported in the table. λref: noncentrality parameter (λ) calculated based on α = 0.05, β = 0.2, and degrees of freedom equal N-1. Sample size is considered appropriate when λ computed on each gene is higher than λref.

## Supplementary Materials

The following are available online at https://www.mdpi.com/2075-4418/9/3/113/s1.
