# Supplementary material for: An Easy and Reliable Strategy for Making Type I Interferon Signature Analysis Comparable among Research Centers
Source: Diagnostics (Basel). 2019 Sep 4;9(3):113. doi: 10.3390/diagnostics9030113 (PMC6787630; doi:10.3390/diagnostics9030113)
Supplement: Supplementary file 1 [file diagnostics-09-00113-s001.pdf]

Supplementary Materials

Table S1. Patient's clinical diagnosis. SAVI: STING-associated vasculopathy with onset in infancy.

Subject n.	Clinical Diagnosis
1	SAVI-like disease
2	Patient's relative
3	Patient's relative
4	SAVI-like disease
5	Patient's relative
6	CANDLE syndrome
7	Sarcoidosis
8	Wiskott Aldrich syndrome
9	Trichohepatoeneteric syndrome 1
10	Dyskeratosis congenita
11	DNase II deficiency
12	CANDLE syndrome
13	CANDLE syndrome
14	AGS-like
15	SLE
16	Copa syndrome
17	Patient's relative
18	Patient's relative
19	IPEX-like syndrome
20	LRBA deficiency

CANDLE: IFN-mediated disease chronic atypical neutrophilic dermatosis with lipodystrophy and elevated temperature, AGS: Aicardi-Goutières syndrome, SLE: Systemic lupus erythematosus, IPEX: Immunodysregulation polyendocrinopathy enteropathy X-linked.

Table S2. Twenty-controls-sized group ISGs expression values.

Control n.	IFI27	IFI44L	IFIT1	ISG15	RSAD2	SIGLEC1
1	0.96	1.46	6.19	43.69	3.10	1.00
2	0.42	1.42	8.00	42.03	3.76	1.77
3	0.36	0.97	5.16	35.46	3.72	0.94
4	0.07	1.12	3.51	21.14	1.71	0.75
5	0.54	1.07	4.50	21.29	0.72	0.68
6	1.00	1.10	5.50	45.11	2.05	2.47
7	0.22	1.16	4.94	33.67	1.55	1.80
8	0.38	0.63	3.48	43.04	1.64	0.33
9	0.52	1.31	3.53	49.16	1.30	1.20
10	0.28	0.79	5.14	27.07	1.76	0.86
11	0.33	0.88	4.90	22.76	2.32	0.68
12	0.33	1.72	4.17	11.75	2.40	1.09
13	0.25	1.20	6.05	14.02	2.55	1.52
14	0.14	0.95	4.10	15.38	1.82	0.98
15	0.38	1.36	6.80	27.00	2.81	1.55
16	0.18	2.02	5.62	16.71	2.90	1.85
17	0.11	2.17	7.63	17.22	4.10	1.30
18	0.13	1.25	5.08	15.56	2.62	1.24
19	0.22	1.12	2.88	34.90	1.88	1.29
20	0.60	1.40	6.13	64.74	4.60	1.08
